# ^18^F-FDG-PET/MRI in the diagnostic work-up of limbic encephalitis

**DOI:** 10.1371/journal.pone.0227906

**Published:** 2020-01-17

**Authors:** Cornelius Deuschl, Theodor Rüber, Leon Ernst, Wolfgang P. Fendler, Julian Kirchner, Christoph Mönninghoff, Ken Herrmann, Carlos M. Quesada, Michael Forsting, Christian E. Elger, Lale Umutlu

**Affiliations:** 1 Institute for Diagnostic and Interventional Radiology and Neuroradiology, University Hospital Essen, University of Duisburg-Essen, Essen, Germany; 2 Department of Epileptology, University of Bonn, Bonn, Germany; 3 Department of Nuclear Medicine, University Hospital Essen, University of Duisburg-Essen, Essen, Germany; 4 Department of Diagnostic and Interventional Radiology, University Duesseldorf, Medical Faculty, Duesseldorf, Germany; 5 Clinic for Neuroradiology, Clemenshospital Muenster, Muenster, Germany; 6 Department of Neurology, University Hospital Essen, University of Duisburg-Essen, Essen, Germany; Ente Ospedaliero Cantonale, SWITZERLAND

## Abstract

**Introduction:**

Limbic encephalitis (LE) is an immune-related, sometimes paraneoplastic process of the central nervous system. Initial diagnosis and treatment are based on the clinical presentation as well as antibody profiles and MRI. This study investigated the diagnostic value of integrated ^18^F-FDG-PET/MRI in the diagnostic work-up of patients with LE for a cerebral and whole-body imaging concept.

**Material and methods:**

Twenty patients with suspected LE were enrolled in this prospective study. All patients underwent a dedicated PET/MRI protocol of the brain as well as the whole-body. Two neuroradiologists, one body radiologist and one nuclear medicine physician performed blinded consensus readings of each corresponding MRI and PET/MRI dataset of the brain and whole-body. Diagnostic confidence was evaluated on a Likert scale.

**Results:**

Based on integrated PET/MRI 19 / 20 patients were found to show morphologic and / or metabolic changes indicative of LE, whereas sole MRI enabled correct identification in 16 / 20 patients. Three patients with negative MRI showed metabolic changes of the limbic system or extra-limbic regions, shifting the diagnosis from (negative) MRI to positive for LE in PET/MRI. Whole-body staging revealed suspected lesions in 2/20 patients, identified by MRI and PET, one confirmed as malignant and one false positive. Diagnostic confidence for cerebral and whole-body imaging reached higher scores for PET/MRI (cerebral: 2.7 and whole body: 4.8) compared to MRI alone (cerebral: 2.4 and whole body: 4.5).

**Conclusion:**

LE diagnosis remains challenging for imaging as it shows only subtle imaging findings in most patients. Nevertheless, based on the simultaneous and combined analysis of morphologic and metabolic data, integrated PET/MRI may enable a dual platform for improved diagnostic confidence and overall detection of LE as well as whole-body imaging for exclusion of paraneoplastic LE.

## Introduction

Limbic encephalitis (LE) is an autoimmune-mediated syndrome, most commonly caused by either infectious or autoimmune diseases. In a minority of cases, LE is caused by an (undetected) tumor in the patients´ body which activates the immune system, also referred to as “paraneoplastic limbic encephalitis”. Diagnosis remains challenging due to its unspecific clinical presentation with loss of short-term memory, mental status changes or general psychiatric symptoms, as not all patients feature temporal lobe seizures [1]. Positive autoimmune antibodies in the cerebrospinal fluid is a common finding in patients with LE and is considered an important criterion for diagnosing LE. In the majority of cases imaging findings in magnetic resonance imaging (MRI) comprise subtle changes, mostly characterized by T2 hyperintensities and volume alterations in mesial temporal structures, making a correct, standardized diagnosis difficult [2, 3]. These volume alterations are typically characterized by increase or loss of hippocampal or amygdala volume [4, 5]. Apart from MRI, ^18^F-fluorodeoxyglucose positron emission tomography (^18^F-FDG-PET) has been shown to facilitate the visualization of changes of the glucose metabolism in mesial temporal structures as well as extra limbic regions and hence support the correct diagnosis of LE [6]. Metabolic information derived from ^18^F-FDG-PET has been shown particularly helpful in patients with indifferent or negative MRI [7, 8]. Aside from these promising results, to date, studies mostly focused either on sole morphological analysis of MRI or sole metabolic analysis of PET data for diagnosis of LE, lacking dedicated studies on the diagnostic value of integrated ^18^F-FDG-PET/MRI. The introduction of integrated PET/MRI scanners has facilitated a new platform for simultaneously acquired and co-registered morphologic and metabolic data, which has been widely used for numerous application fields.

Hence, the aim of this study was to evaluate the diagnostic value of hybrid ^18^F-FDG-PET/MRI for cerebral evaluation and whole-body imaging to diagnose LE and detect / exclude paraneoplastic LE.

## Material and methods

### Patients and inclusion criteria

The study was conducted in accordance with all guidelines set forth by the approving institutional review board. The study was approved by the ethics committee of the University Duisburg-Essen. All patients gave written informed consent before undergoing ^18^F-FDG PET/MRI. Twenty patients (mean age: 38 years, range: 18–76 years, 15 female, 5 male) with suspected LE were included in this prospective study over the time course of 23 months (Table 1).

**Table 1:** Patient data, antibodies, clinical findings.

Pat.	Sex	Age	Antibody	EEG	Limbic symptoms
**1**	f	32	-	left temp.	+
**2**	f	19	-	left temp.	+
**3**	m	75	LGI1 +	right temp.	+
**4**	m	26	LGI1 +	-	+
**5**	m	33	-	left temp.	+
**6**	f	21	Ma2/Ta+	left temp.	+
**7**	f	31	GAD+	left temp.	+
**8**	m	45	GAD+	-	+
**9**	f	29	GAD+	left temp.	+
**10**	f	30	GAD+	bilat.temp.	+
**11**	f	39	GAD+	bilat.temp.	+
**12**	f	37	-	right temp.	+
**13**	f	32	GAD+	left temp.	+
**14**	m	59	GAD +	right temp.	+
**15**	f	20	-	bilat.temp.	+
**16**	f	32	CV2+	left temp.	+
**17**	f	44	CV2+	-	+
**18**	f	76	-	right temp.	+
**19**	f	62	GAD+	-	+
**20**	f	18	-	bilat.temp.	+

Positive antibody profiles were detected in 13 patients (8 with antiglutamic acid decarboxylase (GAD) antibodys, two patients with positive leucine-rich glioma-inactivated 1 (LGI1), two patients for anti-CV2 (CV2) and one patient for anti-Ma2/Ta (Ma2/Ta). 16 patients showed pathologic EEG findings and all patients showed positive limbic symptoms.

Suspected LE was diagnosed by the treating physician based on the German Guidelines on immune related cerebral disease [9]. All patients had suffered from at least one limbic symptom within the last 5 years (e.g. dysfunction of the episodic memory, seizures with temporal semiology, or psychiatric symptoms with affect lability), that initially showed a subacute onset with rapid progression. Moreover at least one of these parameters: (1) positive autoimmune antibodies in the cerebrospinal fluid or (2) brain abnormalities of the medial temporal lobe on T2-weighted MRI restricted to one or both medial temporal lobes (Table 1). Reasonable exclusion of alternate causes. Moreover, the treating physician used all information including electroencephalogram (EEG) findings and therapy response on anti-autoimmune treatment for diagnosis [10].

### Brain PET/MRI

PET/MR Imaging was performed on an integrated 3T hybrid PET/MRI system (Biograph mMR, Siemens Healthcare, Erlangen, Germany). Prior to the examination patients were prepared in accordance to the EANM guidelines for PET brain imaging using ^18^F-FDG [11]. Image acquisition started 60 min intravenous after injection of ^18^F-FDG (mean ± SE, 236.5 MBq; ± 10,04 MBq). PET imaging of the head was performed in one bed position (axial field of view 25.8 cm) with an acquisition time of 20 min while simultaneously acquiring the MRI sequences. PET data were reconstructed in 3D mode using ordinary Poisson ordered subsets expectation maximization with 3 iterations and 21 subsets and a Gaussian filter with 4 mm FWHM and 344 × 344 voxels. For attenuation correction Dixon sequence-based AC was used.

MR imaging was performed simultaneously to PET data acquisition, utilizing a dedicated 16-channel head and neck radiofrequency coil. The MRI protocol comprised the following sequences, detailed information is displayed in Table 2:

**Table 2:** Sequence parameters for the diagnostic MR sequences used in PET/MRI.

Name	Region	Orientation	TR (ms)	TE (ms)	Matrix size	Slice thickness (mm)
**3D-T1-MPRAGE**	Brain	sagittal	1670	2.56	0/320/310/0	0.8
**3D-FLAIR**	Brain	sagittal	5000	284	0/256/248/0	1
**SWI**	Brain	transversal	26	20	0/256/187/0	2
**STIR TSE**	Brain	coronal	5930	25	0/512/384/0	2
**T2-TSE**	Brain	temporal	6000	96	0/256/187/0	4
**T2-TSE**	Brain	ACPC	6000	96	0/512/384/0	4
**ce 3D-MPRAGE**	Brain	sagittal	1790	2.67	0/512/256/0	1
**VIBE**	Whole-body	coronal	3.64	1.49	512/0/0/230	3.5
**DWI**	Whole-body	transversal	6500	56	160/0/0/90	5
**HASTE**	Whole-body	transversal	1500	97	320/0/0/194	7
**ce VIBE**	Whole-body	transversal	3.64	1.49	512/0/0/230	3.5

1. a high-resolution three-dimensional (3D) magnetization-prepared rapid acquisition with gradient echo (3D-T1-MPRAGE) sequence
2. a three-dimensional (3D) fluid-attenuated inversion recovery (3D-FLAIR) sequence
3. a transversal susceptibility-weighted imaging [12] sequence
4. a coronal Short tau inversion recovery (STIR) sequence
5. a temporal and an anterior commissure—posterior commissure orientation (ACPC) T2 weighted turbo spinecho sequence
6. a contrast-enhanced high-resolution three-dimensional (3D) magnetization-prepared rapid acquisition with gradient echo (ce3D-T1-MPRAGE) sequence (0.2 mmol/1 kg body weight of contrast agent (Dotarem®, Guerbet, Sulzbach/Taunus, Germany)).

### Whole-body PET/MRI

After the brain examination whole-body imaging was obtained in 4–5 bed positions (from skull-base to mid-thighs) with a PET acquisition time of 4 min / bed position. PET image reconstruction was performed subsequently utilizing the OSEM algorithm, 3 iterations and 21 subsets, a Gaussian filter with 4 mm, FWHM and a 344x344 image matrix. For MRI data acquisition a dedicated mMR head-and-neck radiofrequency (RF) coil and RF body array surface coils were used [13]. The total scan duration of the whole-body protocol amounted to 27.8±3.7 min and comprised the following sequences, detailed information is displayed in Table 2:

1. a 3-dimensional volume interpolated breath-hold examination (VIBE) sequence for Dixon-based attenuation correction
2. a diffusion-weighted (DWI) echo-planar imaging (EPI) sequence
3. a 2-dimensional half Fourier acquisition single-shot turbo spin echo (HASTE) sequence
4. a post-contrast 3-dimensional fat-saturated VIBE sequence.

### Image analysis

Two board certified neuroradiologists, one board certified body radiologist and one board certified nuclear medicine physician performed consensus readings of the MRI, PET and fused PET/MRI datasets of the brain and whole-body. MRI and PET/MRI were assessed independently in two sessions with a break of 4 weeks to avoid recognition bias. Readings were performed on a PACS workstation (Centricity Radiology R1000, GE Healthcare, IL, USA) as well as on a dedicated viewing software for integrated imaging (Scenium Software, Syngo.via; Siemens Healthcare, Erlangen, Germany). The readers were blinded for the patients´ final diagnosis but had access to all relevant clinical information like EEG, clinical presentation, antibody status.

#### Brain imaging

All datasets were visually interpreted. MR-criteria indicative of a limbic encephalitis include (1) T2 hyperintensity of the amygdala, of the hippocampus and/ or insula and (2) volume alterations of the mesial temporal structures [2, 14]. Additionally, PET images were visually interpreted and analysed for any hypo- or hypermetabolism of the limbic system. Aside from changes in the limbic system, any extra-limbic metabolic changes indicative / potentially associated to LE were noted as well. Furthermore, the readers used stereotactic surface projection for analysis (SSP, Syngovia, Siemens Healthcare, Erlangen, Germany). SSP analysis was performed based on a software implemented matched reference cohort scanned on a Biograph PET/CT supplied by SyngoVia.

In addition, the readers indicated their diagnostic confidence on a Likert scale (1-not at all confident, 2-not very confident, 3-neutral, 4-confident, 5-very confident) for MRI alone, PET alone and integrated PET/MRI for brain imaging. Data are presented as mean +/- standard deviation. Descriptive analysis was used to evaluate the resulting scores.

#### Whole-body imaging

In accordance with previous publications the following criteria were considered indicative of malignancy of lesions in the whole-body datasets: (1) lesion shape, (2) local invasiveness, (3) central necrosis, (4) increased contrast enhancement, and (5) diffusion restriction [15]. Lymph nodes with a short axis diameter > 10 mm, spherical configuration and increased contrast enhancement were classified as nodal metastases. In accordance with previous publications visually detectable focal ^18^F-FDG uptake above the surrounding background was considered as a sign of malignancy on PET / PET/MRI [15].

## Results

Hybrid ^18^F-FDG-PET/MRI was successfully completed in all 20 examinations and no relevant artifacts (leading to an exclusion of datasets) were detected.

### Brain imaging

#### Morphologic imaging

Two board certified neuroradiologists performed a consensus reading of the MRI datasets and reported changes of the limbic system in 16/20 patients (80%) (Table 3).

**Table 3:** Results of cerebral PET/MRI.

Pat.	Antibody	MRI	MRI Finding	PET	PET Finding	Z-Scores *
**1**	-	+	Amygdala and hippocampus swelling and T2 hyperintensity left	+	Bifrontal hypometabolsim	1.1; 1.2 // 2; 1.9 // -0.5; 0.7 // 1.4; 1.9 // 1.6; 1.6
**2**	-	+	Hippocampal sclerosis left	+	Hypometabolism left amygdala	-0.1; -1.8 // 0.8; -0.1 // -2.5; -3.8 // -0.6; -1.6// 0; 0.1
**3**	LGI1 +	+	Bilateral amygdala swelling and T2 hyperintensity	+	Hypermetabolism right amygdala and hippocampus & bitemporal, -and biparietal and hypometabolism	4.9; 2.4 // 3.3; 1.8 // -1.1; -0.9 // -0.3; -0.8 // 1.3; 0.7
**4**	LGI1 +	-	-	-	-	-1.2; 0.2 // -0.3; -0.4 // -2.4; -2 // -1.1; -1.2 // 0.2; 0
**5**	-	-	-	+	Hypometabolism left amygdala	1; -2// 0.1; -0.4 // -0.9; -1.7 // -0.4; -0.5 // -1; -0.8
**6**	Ma2/Ta+	+	Amygdala swelling an T2 hyperintensity left	+	Hypometabolism left amygdala, hippocampus & bitemporal and biparietal hypometabolism	0.2; -1.8 // 0.4; -0.2 // -1.5; -3.8 // -0.6; -1.3 // 0.1; 0
**7**	GAD+	-	-	+	Hypometabolism left amygdala + bitemporal and biparietal lobe hypometabolism	-1.2; -2.5 // -0.7; -0.9 // -3.3; -2.9 // -2.3; -1.8 // 1.4; 1.3
**8**	GAD+	+	Amygdala and hippocampus swelling an T2 hyperintensity left	-	-	-0.4; -0.5 // -0.9; -2.3 // 1.4; 1.9 // 1.3; 1.8 // -2.1; -1.6
**9**	GAD+	+	Amygdala swelling and T2 hyperintensity left	-	-	0.2; 0.3 // 0.2; -0.4 // 1.1; 0.5 // 1.3; 0.8 // -0.7; -1.5
**10**	GAD+	+	Amygdala swelling and T2 hyperintensity bilateral	-	-	0.5; -0.4 // -2.3; -0.9 // 1.9; 1.4 // 2.8; 1.3 // -2.1; -1.4
**11**	GAD+	+	Amygdala swelling and T2 hyperintensity left	-	-	1.8; 1.1 // 1; 1.3 // 0; -0.3 // -0.4; -0.4 // 1; 0.8
**12**	-	-	-	+	Bitemporal and biparietal hypometabolism	1.1; 0.5 // 1.1; 1.5 // -0.9; -0.7 // 0.4; 0.6 // 1.1; 1.1
**13**	GAD+	+	Hippocampus and amygdala T2 hyperintensity bilateral	-	-	-0.5; 0 // -0.9; -0.1 // -2.2; -1.9 // -2; -1.2 // -1; -0.5
**14**	GAD +	+	Hippocampus swelling and amygdala T2 hyperintensity right	+	Hypometabolism right amygdala	-1.1; 1.2 // 0.9; 1.6 // -0.1; -0.5 // 0.4; -0.5 // 0.5; 0.6
**15**	-	+	Hippocampus and amygdala T2 hyperintensity bilateral	-	-	0.7; 0 // -0.7; -0.3 // -0.8; -1.7 // -0.3; -0.8 // -1.3; -1.1
**16**	CV2+	+	Hippocampus and amygdala T2 hyperintensity bilateral	-	-	0.2; -0.1 // -0.5; 0.4 // -3.4; -2 // -1.1; 0 // -0.4; -0.1
**17**	CV2+	+	Hippocampus and amygdala T2 hyperintensity bilateral	-	-	0.6; 0.2 // -0.6; 0.5 // 1,1; -0.6 // 0.6; 0 // 0.6; 1.2
**18**	-	+	Hippocampus and temporal lobe swelling and T2 hyperintensity right	+	Hypometabolism right amygdala and hippocampus & hypometabolism right parietal lobe	-1.2; -0.8 // 0.5; -0,6 // -0.6; -1.1 // 0; 0.6 // 1.2; 0.6
**19**	GAD+	+	Hippocampus and amygdala swelling and T2 hyperintensity bilateral	+	Hypometabolism right amygdala	-1.6; 0.2// -0.6; 0.5// -1.1; -0.6 // 0; 0.6 // 0.6; 1.2
**20**	-	+	Amygdala swelling and T2 hyperintensity left	-	-	0.1; 1 // 1.2; 1.1 // -2; -1.6 // -2.4; -2.7 // -1.6; -1.1

*Z-Scores from detailed SSP analysis displayed as following: Amygdala right; amygdala left // hippocampus right; hippocampus left // temporal pole: superior temporal gyrus right; temporal pole: superior temporal gyrus left // temporal pole: middle temporal gyrus right; temporal pole: middle temporal gyrus left // thalamus right; thalamus left.

**Fig 1:**
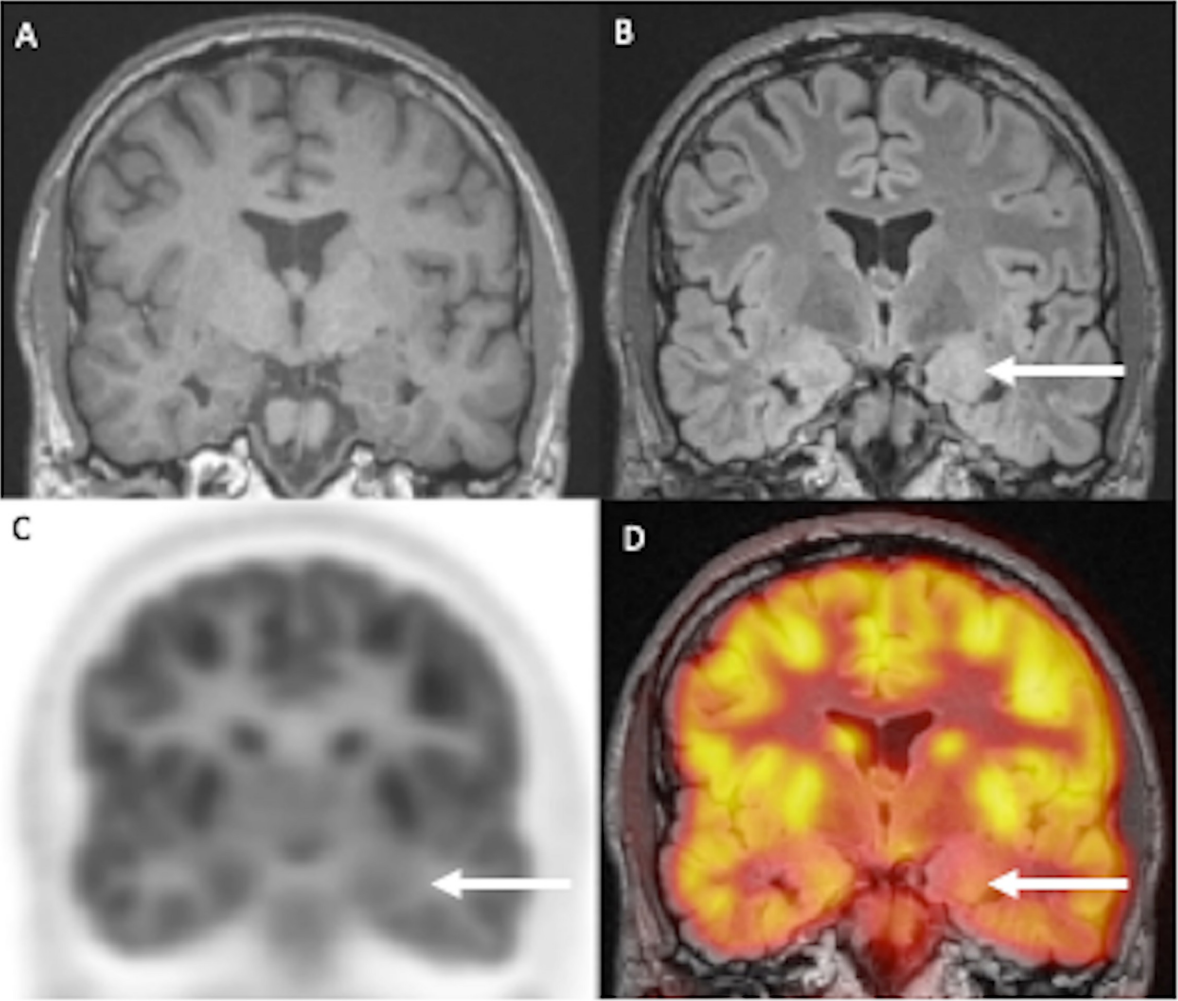
21-year old female patient with swelling and FLAIR hyperintensity of the left amygdala (A, B). PET shows corresponding glucose hypometabolism (C, D).

**Fig 2:**
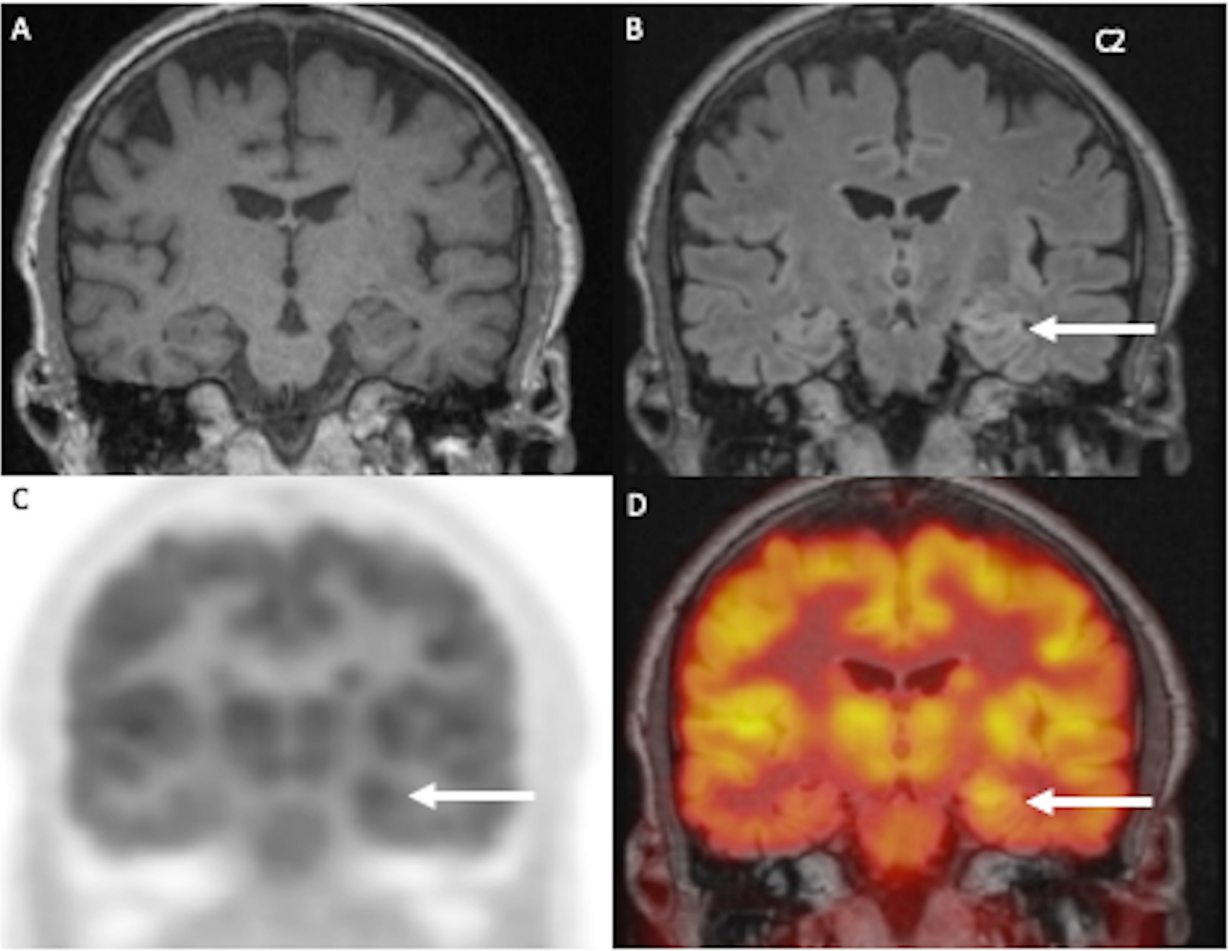
75-year old patient with left sided enlargement of the hippocampus on T1 (A) and dull FLAIR-hyperintensity (B). Corresponding glucose hypermetabolism of the left hippocampus and amygdala can be detected on FDG-PET (C) and fusion with FLAIRw (D).

Out of these 16 patients, 8 showed unilateral enlargement and/or T2/FLAIR signal increase of the amygdala (Figs 1A, 1B, 2A and 2B) and the hippocampus.

7 patients demonstrated bilateral swelling and T2/FLAIR signal increase of the amygdala and/or hippocampus. In one patient hippocampal sclerosis was detected based on a reduced hippocampal volume with increased T2/FLAIR signal. In 4/20 patients no pathologic changes were found on MRI.

#### Hybrid imaging

One board certified nuclear medicine physician and two board certified neuroradiologists analyzed the PET/MRI datasets of the brain in a consensus reading. The evaluation of hybrid imaging revealed corresponding morphologic and or metabolic changes of the limbic system and extra limbic areas in 19/20 patients (95%). Out of these 19 patients, 16 patients showed morphologic changes, whereas 7 of these patients showed additional metabolic changes (three with changes of the limbic system, three with changes of the limbic system and additional extra-limbic involvement and one patient with isolated extra-limbic hypometabolism) (Figs 1 and 2). Three of the 20 patients showed negative MRI but positive PET, one revealing metabolic changes of the limbic system, one with hypometabolism of the limbic system and the extra-limbic structures and one patient with extra-limbic involvement, changing the diagnosis from negative MRI to suspected LE in hybrid imaging. Another patient did not show any pathological changes in MRI or PET (Table 3).

#### Diagnostic confidence

Diagnostic confidence on a five-point Likert scale reached higher values for hybrid PET/MRI with 2.7 when compared to sole MRI with 2.4.

### Whole-body imaging

#### Morphologic imaging

One board certified body radiologist and a nuclear medicine specialist performed a consensus reading of the whole-body MRI sequences. In 2/20 patients suspicious lesions were detected (Table 4).

**Table 4:** Results of whole-body PET/MRI.

Patient #	Whole-body MRI	Whole-body PET	Whole-body diagnosis
**1**	-	-	
**2**	-	-	
**3**	+	+	rectal cancer
**4**	-	-	
**5**	-	-	
**6**	-	-	
**7**	-	-	
**8**	-	-	
**9**	-	-	
**10**	-	-	
**11**	-	-	
**12**	-	-	
**13**	-	-	
**14**	+	+	false positive
**15**	-	-	
**16**	-	-	
**17**	-	-	
**18**	-	-	
**19**	-	-	
**20**	-	-	

**Fig 3:**
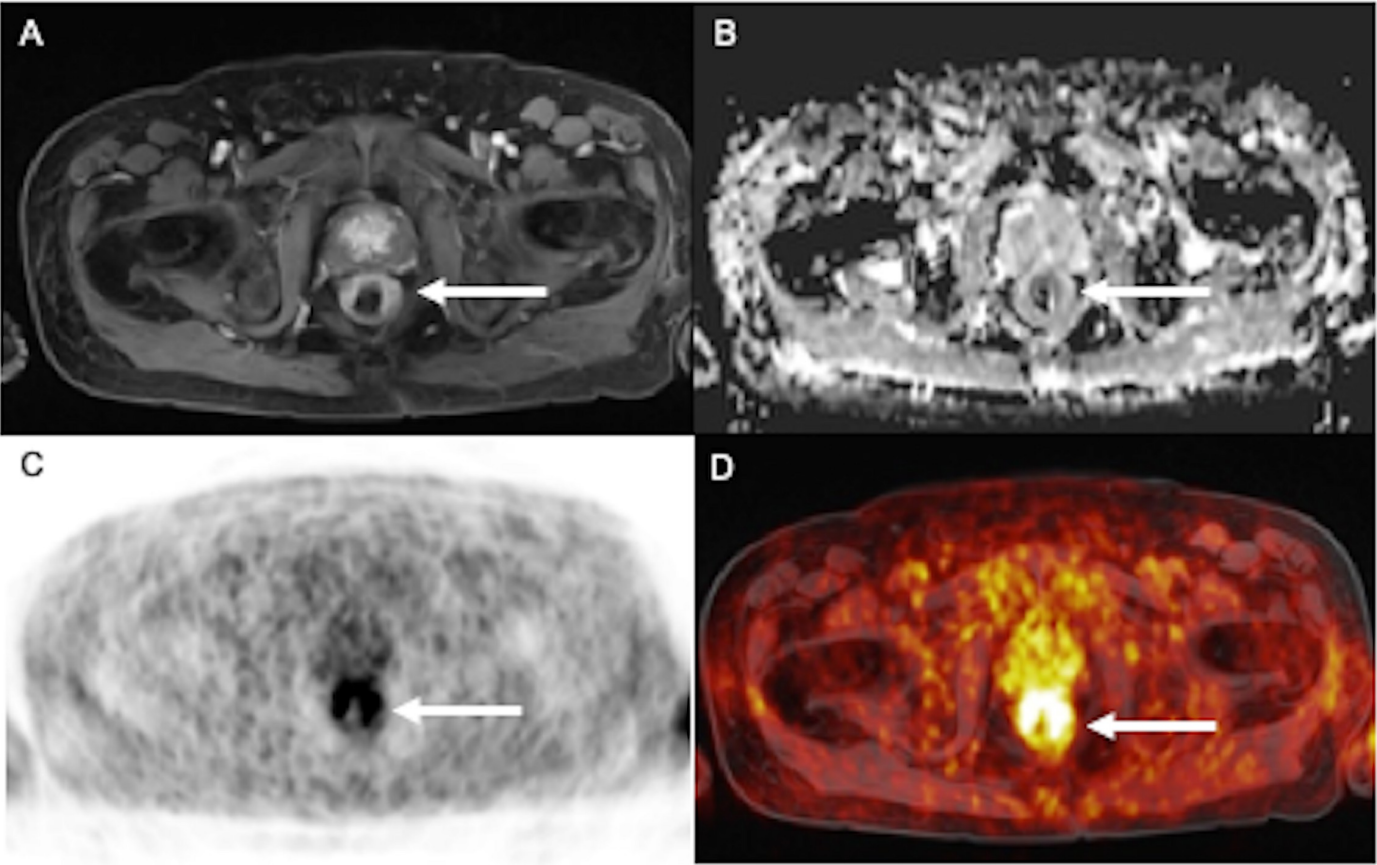
75-year old patient with circumscribed thickening of the rectal wall and dull contrast enhancement (A), with a distinct diffusion restriction on the ADC-MAP (B). PET shows an intense semicircular tracer uptake with SUVmax of 8.8 (C, D). Final histopathological diagnosis was a rectal cancer.

In one patient rectal cancer was diagnosed on MRI and confirmed by histopathology (Fig 3).

**Fig 4:**
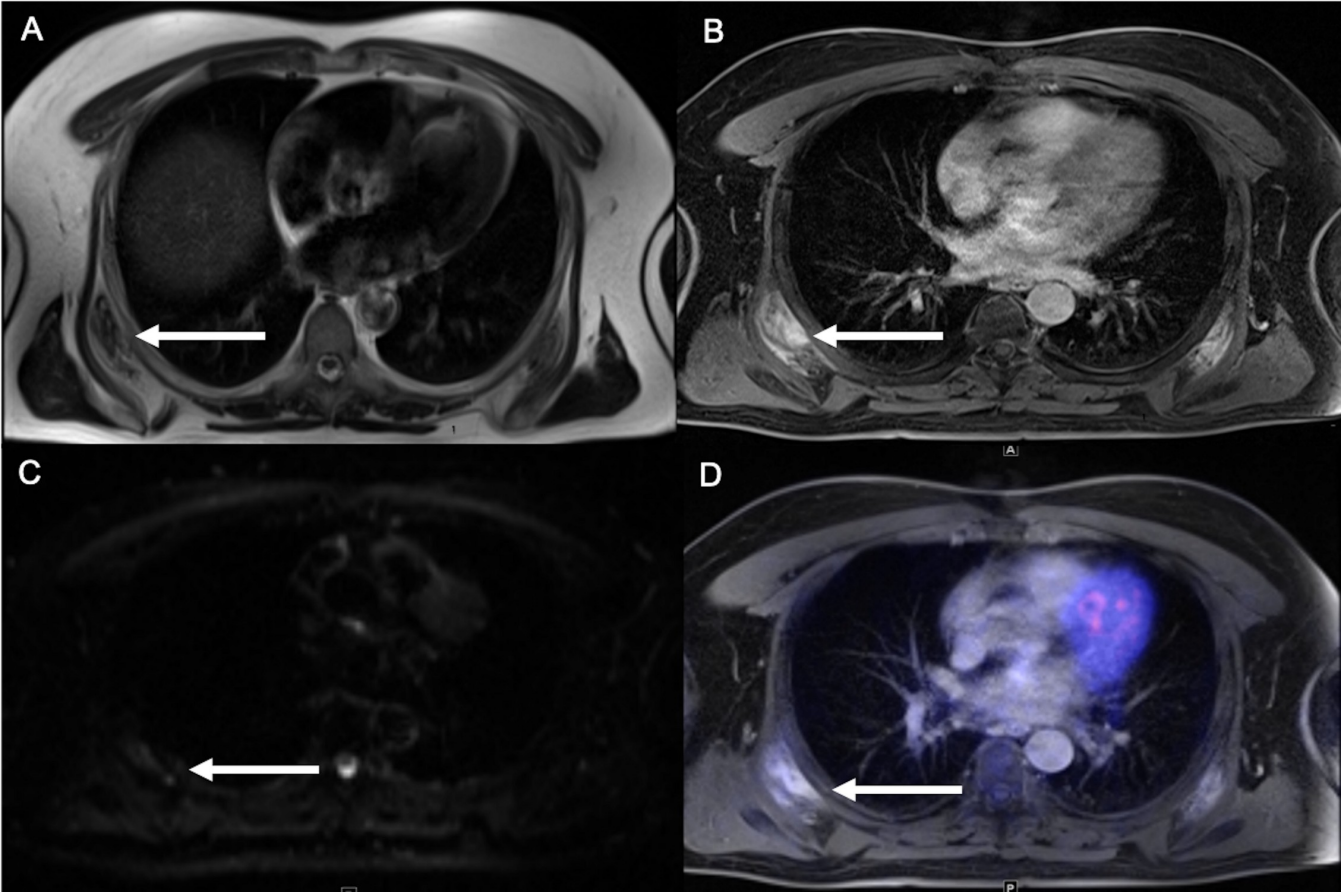
59-year old patient with suspicious subscapular lesions with isointense signal on T2w (A), intense inhomogeneous contrast enhancement (B), no diffusion restriction (C) and mild tracer uptake with SUVmax of 3.2 (D).

In one patient suspicious bilateral subscapular lesions were described on MRI with mild glucose uptake (Fig 4).

Further clinical diagnostics showed no evidence of oncological disease in this patient, making an elastofibroma dorsi the most likely diagnosis [16].

#### Hybrid imaging

One board certified body radiologists and a nuclear medicine specialist performed a consensus reading of the whole-body PET/MRI sequences (Table 4). Comparable to the MRI datasets, two suspicious lesions with pathologic SUVs were detected. Further diagnostic work-up revealed the correct identification of one patient with rectal cancer and one false positive diagnosis (inflammatory subcutaneous lesion versus tumorous).

#### Diagnostic confidence

Diagnostic confidence for whole-body staging reached higher values for hybrid PET/MRI with 4.8 when compared to sole MRI with 4.5.

## Discussion

Our study results on ^18^F-FDG PET/MRI in the diagnostic work-up of limbic encephalitis deliver three important findings: First, hybrid ^18^F-FDG-PET/MRI identified more patients with LE compared to MRI alone. Second, hybrid ^18^F-FDG-PET/MRI scored higher diagnostic confidence to identify LE compared to MRI alone. Overall, both aspects underline the importance and additional diagnostic value of ^18^F-FDG to MRI, providing complementary information for an improved diagnosis of LE patients.

LE experienced growing recognition within the last years as a rare cause of altered mental status [17, 18]. Diagnosis is often missed or delayed due to an unspecific clinical presentation with limbic dysfunction as the single most consistent finding [19]. Diagnosis of LE is based on imaging in addition to clinical presentation with limbic symptoms, specific antibody subtypes and EEG patterns [9]. Imaging of LE is mainly based on MRI and analysis of FLAIR and T2-w images, commonly comprising volume alterations and swelling-induced signal increase on T2-w and FLAIR images of the limbic system [2, 3, 14, 20]. Nevertheless, a variety of imaging findings have been reported for extra-limbic areas, including the frontal or parietal cortices [21–24], the cerebellum or the brain stem [8, 25, 26]. Showing a great variety of occurrence of pathological findings in MRI, ranging from less than 10% to 100% [8, 23], MRI has been demonstrated to yield false negative results in a vast number of patients [2]. While no direct correlation to influencing factors has been identified that could explain these highly variable imaging findings, there seems to be an association to specific subtypes such as the group of II anti-N-methyl-D-aspartate (NMDA) receptor and VGKC (Voltage-gated potassium channel)-associated encephalitis [2, 27, 28].

In addition to cerebral MRI whole-body ^18^F-FDG-PET is often performed for tumor screening in LE patients to rule out paraneoplastic limbic encephalitis and has been shown to be highly sensitive for the detection of epileptogenic lesions [29, 30] and may also support the diagnosis of LE. To date, most ^18^F-FDG-PET publications describe changes in the mesial temporal metabolism, often accompanied by a variety of further metabolic changes in the associating cortex as well as relative metabolic sparing of primary cortices, cerebellum and striatum [8, 31–34]. Nevertheless, comparable to the wide range of variable changes in MRI, ^18^F-FDG-PET has also been reported to yield varying metabolic changes from hyper- and hypometabolism involving various regions of the brain to completely normal ^18^F-FDG-PET scans [8, 31, 35]. A number of studies indicate that these PET findings (in the limbic system and extra-limbic regions) were associated with clinical symptoms and active disease status more strongly than the MRI findings [7, 36]. A recent Editorial emphasized the importance of harmonized brain ^18^F-FDG-PET protocols, making PET findings consistent and comparable between different centers [37]. Combining the strength of both MRI and PET in a single temporal as well as spatial domain, as well as facilitating a one-stop-shop examination, by means of cerebral and whole-body imaging, the aim of our study was to investigate the according morphologic and metabolic changes in patients with LE while ruling out potential causes for paraneoplastic limbic encephalitis. To date, most studies on hybrid imaging in LE put the focus on the sole investigation of PET or MRI, with only a few case reports describing the utilization of integrated PET/MRI for LE diagnostics [38]. Our results confirm previous studies in demonstrating a great variety of morphologic and metabolic changes in patients with LE in the limbic system and extra limbic areas as well as the importance and great diagnostic value of adding ^18^F-FDG-PET to the morphologic assessment. Overall, MRI showed high diagnostic accuracy for the correct identification of LE with 16/20 patients. PET was negative in 9 of these patients, and positive in metabolic changes of the limbic system and/or extra limbic areas in 7 patients underlining the previously published variability of involved brain regions (Table 3). These results go inline with observations by Probasco et al. suggesting that autoimmune encephalitis may involve broader metabolic abnormalities detectable by ^18^F-FDG-PET in extra limbic areas of the brain [39]. In accordance with previous publications, the vast majority of patients showed hypometabolism in our study with only one patient showing hypermetabolism of the limbic system (Table 3) [8, 40]. Apart from improving the diagnostic confidence, the added PET data also helped to correctly identify patients with negative MRI, in revealing metabolic changes of the limbic system in two patients and of the extra limbic system in one more patient. Overall, the results of our study and previous publications indicate that ^18^F-FDG-PET may facilitate complementary information to morphologic MR features and may represent a sensitive and early biomarker for LE [39]. Nevertheless, the rather low scoring rates of diagnostic confidence of brain PET/MRI for detection of LE when compared to body PET/MRI clearly illustrate the remaining and outstandingly high challenges of imaging based LE diagnosis.

Apart from facilitating the dual assessment of cerebral morphologic and metabolic data, integrated PET/MRI also enables dual assessment of cerebral and whole-body imaging. With tumor-induced paraneoplastic limbic encephalitis being one of the three major causes of limbic encephalitis, patients suspected of LE commonly undergo whole-body imaging to rule out underlying oncologic diseases. As reported in a vast amount of previous publications integrated PET/MRI facilitates a highly valuable platform for whole-body staging [15, 41–43]. In our study, an underlying oncologic disease could be ruled out in 18 patients, while one patient with rectal cancer could be correctly identified, and one suspicious subcutaneous lesion in another patient was false positive in MRI as well as PET.

Overall, our study is not free of limitations. The clinical setting of the study resulted in the inclusion of a heterogeneous cohort of a minority of treated and majority of untreated patients. Although no direct interdependency between treatment and PET/MRI analysis can be concluded, this issue of heterogeneity and the small patient cohort should be addressed in future trials comprising larger patient cohorts.

Overall, our study results underline the importance and diagnostic value of the combined morphologic and metabolic assessment for LE diagnostics. Furthermore, they demonstrate the manifold benefit of integrated PET/MRI systems in facilitating not only a simultaneous analysis of MRI and PET, but also of cerebral and whole-body data.
